# Anthropometric Parameters and Mediterranean Diet Adherence in Preschool Children in Split-Dalmatia County, Croatia—Are They Related?

**DOI:** 10.3390/nu13124252

**Published:** 2021-11-26

**Authors:** Dora Bučan Nenadić, Ela Kolak, Marija Selak, Matea Smoljo, Josipa Radić, Marijana Vučković, Bruna Dropuljić, Tanja Pijerov, Dora Babić Cikoš

**Affiliations:** 1 Department of Nutrition and Dietetics, University Hospital Centre Split, 21000 Split, Croatia; marija.selak3@gmail.com; 2 Croatian Association of Nutritionists, 10000 Zagreb, Croatia; matea.cigic@gmail.com (M.S.); brunatripicic@gmail.com (B.D.); pijerovtanja@gmail.com (T.P.); dorababic1@gmail.com (D.B.C.); 3 Department of Nephrology and Dialysis, University Hospital Centre Split, 21000 Split, Croatia; josiparadic1973@gmail.com (J.R.); mavuckovic@kbsplit.hr (M.V.); 4 Department of Internal Medicine, University of Split School of Medicine, 21000 Split, Croatia

**Keywords:** preschool children, nutritional status, eating habits, Mediterranean diet, Split-Dalmatia County

## Abstract

Obesity is a rapidly growing problem in European countries, Croatia being among them. According to the latest CroCOSI data, every third child in Croatia aged 8.0–8.9 years is overweight or obese. The Mediterranean diet (MeDi) and its impact on nutritional status and health has been the focus of recent research. Therefore, the aim of this cross-sectional, observational study was to determine the nutritional status and adherence to the MeDi of preschool children in Split, Croatia. We included 598 preschool children aged 3 to 7 years and, for each child, parents completed a lifestyle questionnaire and the Mediterranean Diet Quality Index (KIDMED) in order to assess adherence to the MeDi. The anthropometric assessment included the measurement of weight, height, mid-upper arm circumference (MUAC), waist circumference (WC) and the z-score was calculated. According to the z-score, 420 (70.2%) children had a healthy body weight with 54 (9%) underweight and 124 (20.8%) overweight or obese children. Almost half (49%) of the study participants had a low KIDMED index score, indicating a low MeDi adherence, 37% had an average score, while only 14% had high MeDi compliance. Statistically significant negative correlations between MUAC and WC and the consumption of a second daily serving of fruit (*p* = 0.04) as well as a daily serving of vegetables (*p* = 0.03) were found. In conclusion, low compliance to the MeDi principles in preschool children is concerning. Considering the beneficial effects of the MeDi on overall health, further education, and the adoption of healthy eating habits in preschool children in this Mediterranean region are required.

## 1. Introduction

For preschool children, a healthy nourishment characterized by an adequate and balanced nutritional intake alone can ensure proper physical growth as well as cognitive and emotional development, a feeling of satiety and sufficient physical ability [1]. In this preschool period, children shape their dietary patterns by developing independent feeding skills and forming their own food preferences [2]. In addition to the direct effect on growth and development, inadequate nutrition can affect the occurrence of certain chronic non-communicable diseases in childhood such as cardiovascular diseases, obstructive sleep apnea and psychosocial problems [3]. Given that eating habits acquired in childhood might continue into adulthood [4] and increase the risk of the development of type 2 diabetes mellitus, arterial hypertension and arteriosclerosis especially in children with persistent obesity [5,6], it is important to provide children with quality nutrition and allow them to adopt proper eating habits [1]. On the other hand, overnutrition, which leads to children becoming overweight and obese because of their excessive intake of energy dense food and a sedentary lifestyle, is a rapidly growing problem in European countries [3] and as such in Croatia. According to the European Childhood Obesity Surveillance Initiative in Croatia (CroCOSI, 2018–2019), one in three Croatian children (35.0%) aged 8.0–8.9 years were overweight and obese [7]. In the past few years, nutrition research has focused on the MeDi and its impact on nutritional status and health. Considered as one of the healthiest dietary patterns, MeDi is based on high intake of fresh fruits, vegetables, legumes, whole grains, dairy products, fish and olive oil; moderate intake of poultry, eggs and wine, and finally, a low intake of red and processed meat and sweets [8]. Greater adherence to the MeDi is associated with a significant reduction of risk in developing chronic morbidities [9] and an inverse relationship with obesity [10]. In order to assess the compliance to the principles of the MeDi in the pediatric population, the use of the KIDMED index is suggested [11]. As the growth and nutritional assessment by health professionals become less frequent after the first year of a child’s life, dietary habits are most often guided by parents, caregivers, or kindergarten professionals. Therefore, the nutritional status and dietary habits of preschool children should be given special attention. A departure from the traditional MeDi has been observed in the general population [12], and according to recent research the same drift has occurred in preschool children [13]. Therefore, the main aim of this study was to determine nutritional status and adherence to the MeDi in preschool children from Split-Dalmatia County, Croatia. 

## 2. Materials and Methods

### 2.1. Study Design and Population

Five hundred and ninety-eight preschool children aged 3 to 7 years were included in this cross-sectional study. The study was carried out in randomly chosen kindergartens in Split, Split-Dalmatia County, Croatia between March and October 2019. The research was conducted as part of a series of lectures for educators and parents as well as educational workshops for children about healthy eating habits for this population at the initiative of the Croatian Association of Nutritionists. Oral and written informed consent was obtained from parents of each study participant as well as from kindergarten management. Only one child per household was included in the study. Children who met one of the following criteria were excluded from the study: (1) refused to participate; (2) had psychological disorders that prevent them from participating in regular activities; (3) lack of parental approval; (4) in cases where parents had not fully completed or returned questionnaires. 

### 2.2. Lifestyle Questionnaire

Parents who had consented to their child participating in the study were asked to complete a lifestyle questionnaire to provide insight into the characteristics of the population. The questionnaire included demographic information such as date of birth and sex as well as questions about the duration of stay at the kindergarten, possible present food allergies, number of meals and the use of dietary supplements. 

### 2.3. The Mediterranean Diet Quality Index (KIDMED) 

For each study participant, parents were also asked to complete the KIDMED index. The KIDMED index was created to assess adherence to the MeDi for children and young people from two (2) to twenty four (24) years of age based on the principles that reflect Mediterranean eating habits and those that undermine them [14]. The index itself consists of sixteen questions that can be answered with the response of yes or no. Questions with negative connotations were assigned a value of −1 and those with a positive connotation were attributed a value of +1 [15]. The sum of all values range from zero (0) to twelve (12) and are therefore classified into the following three levels: >8 which indicates the optimal MeDi; 4–7, which means adjustment is needed to improve food intake according to the MeDi principles; ≤3 i.e., very low quality of nutrition according to the MeDi [11]. 

### 2.4. Anthropometric Measurements 

Each child underwent an anthropometric assessment at the kindergarten while wearing light clothes. Height was measured using a stadiometer and weight was measured to the nearest tenth decimal with a calibrated Omron BF511 diagnostic scale (Omron, Kyoto, Japan). Non-stretchable, flexible body-measuring tape was used to measure the mid-upper arm circumference (MUAC) and waist circumference (WC). For each study participant, a BMI-to-age z-score was calculated with WHO AnthroPlus software [16]. Because of its ability to describe nutritional status, including at the extreme ends of the distribution and the ability to derive summary statistics, this classification system is recommended by WHO (for, i.e., means and SDs of z-scores (WHO, 1995)). Z-scores are derived using the exact age in days for the WHO standards and months for the WHO reference of 2007 [16]. Anthropometric measurements were performed in a playful manner to lessen the anxiety levels of children. No child was forced to participate in anthropometric measuring if unwilling, regardless of parental consent. 

### 2.5. Statistical Analysis

A database was created with the information obtained from the questionnaires and anthropometric assessment were produced for a statistical analysis using IBM SPSS Statistics for Windows, Version 21.0 (IBM Corp, Armonk, NY, USA) software. The categorical data are represented by absolute and relative frequencies (*n* and %). Numerical data were described by arithmetic mean and standard deviation (SD) in cases of normal distribution following, and, in other cases, median and interquartile range boundaries were used. The variance of the category variables was tested by the Chi-square test. A point-biserial correlation was used to measure the strength and direction of the association that exists between one continuous variable and one dichotomous variable. The significance level was set at *p* < 0.05, with a 95% confidence level. 

## 3. Results

Out of the 598 children involved in this study, 310 (51.8%) were boys with a median weight of 21.2 kg. According to the z-score, 420 (70.2%) children had healthy body weight with 54 (9%) children found to be underweight and 124 (20.8%) found to be overweight or obese. The basic characteristics and anthropometric measurements of the study population are shown in Table 1. Nearly 90% of participants were enrolled in a full day kindergarten programme which provides two main meals (breakfast and lunch) as well as morning and afternoon snacks. An example of a menu from kindergartens in which the research was conducted is shown in Table S1.

**Figure 1:**
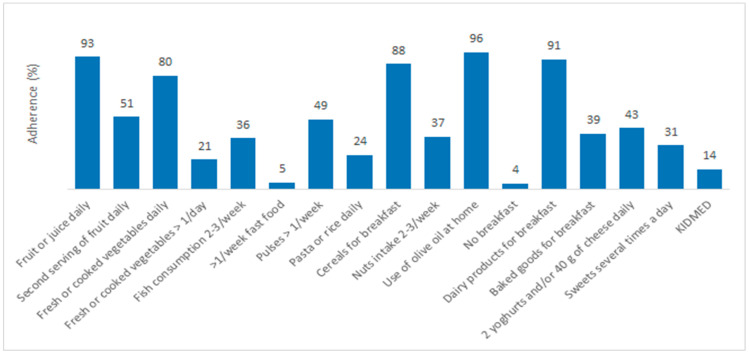
Adherence to the MeDi and its components for total study population.

Almost half (49%) of the study participants had a low KIDMED score, suggesting low MeDi adherence; 37% had an average score while only 14% had high MeDi compliance. The highest compliance to the KIDMED components was determined related to points distributed for eating breakfast, the consumption of dairy products for breakfast, the daily intake of fruit and juice and eating fast food less than once a week. The total adherence to the KIDMED score and its components is shown in Figure 1.

**Figure 2:**
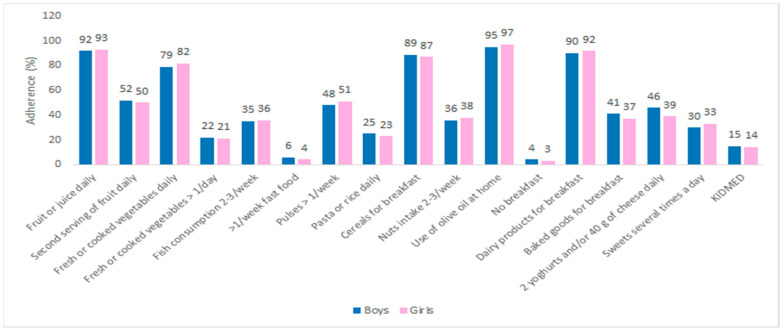
Adherence to the MeDi and its components regarding sex.

There were no statistically significant differences in total KIDMED score regarding sex (Figure 2) nor its components.

**Figure 3:**
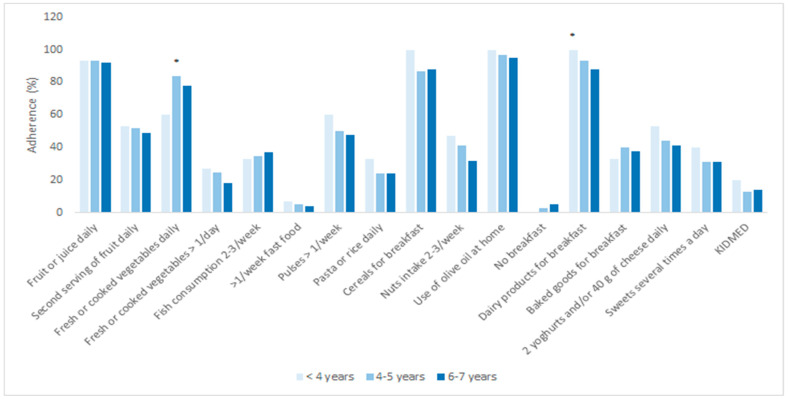
Adherence to the MeDi and its components regarding age. * *p* < 0.05; *p*-values were obtained with Chi-square test.

The most apparent differences with respect to age in responses to the KIDMED questionnaire were found for the consumption of vegetables once a day (*p* < 0.02) and dairy product consumption for breakfast (*p* = 0.04). There was no statistically significant difference between the total KIDMED score and age groups, as demonstrated in Figure 3.

**Figure 4:**
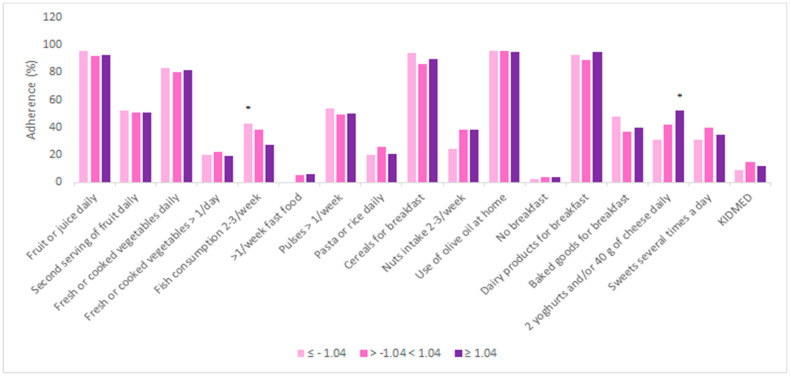
Adherence to the MeDi and its components regarding z-score. * *p* < 0.05; *p-*values were obtained with Chi-square test.

Differences in adherence to the MeDi, according to the nutritional status are shown in Figure 4. Statistically significant differences were not found except for a borderline significance in fish consumption two or three times a week (*p* < 0.04) for undernourished children, and dairy product consumption with a frequency of two or more times a day (*p* < 0.03) in obese children.

A correlation between MUAC and WC and components of the KIDMED index was observed. As shown in Table 2, children who consumed a second daily serving of fruit (*p* = 0.04) as well as a daily serving of vegetables (*p* = 0.03) had a significantly smaller WC, whereas children who had a vegetable intake of more than once a day (*p* = 0.01) had a significantly smaller MUAC.

## 4. Discussion

Out of total study participants, 20.8% were overweight or obese, which was expected considering the increase in the prevalence of obesity in the pediatric population globally and in Croatia. Data from the World Health Organization indicate that approximately 32.8 million children under the age of 5 were overweight or obese in 2019 [17]. According to the latest results of the CROCosi survey from 2019, the percentage of children who are overweight or obese is 36.9%—the highest in the Adriatic region, and 36.9%, of which 23.1% of children were overweight and 13.8% were obese [7]. Almost half of the preschool children included in this study presented a low adherence to the MeDi, underlining low adherence in the general population. These results are in accordance with the study from Kolčić et al. conducted with adults, in which only 23% of the participants from Southern Dalmatia adhered to the principles of the MeDi [12]. As shown in Figure 1, a low intake of fish, nuts, and dairy products most likely contributes to the poor adherence to the MeDi in children, as observed in this study. In previous studies, the adherence to the MeDi, calculated using the KIDMED index, has shown poor results among children [18] and adolescents [19] from the European Union, suggesting the necessity of nutritional intervention in this particular population. On the contrary, the results of an earlier study involving preschool children from Split-Dalmatia County indicate an exceptionally high adherence to the MeDi with only 6% out of 260 children having a low KIDMED score [20]. The difference in the results can be explained by the smaller number of respondents in the above-mentioned study, as well as parental overestimation or underestimation of the quality of a child’s nutrition.

Our results did not show a significant difference in adherence to the MeDi between girls and boys, which is consistent with the results of previous studies from Spain and Turkey [21,22].

A difference in the total KIDMED score with regard to age was not found in our study, but a statistically higher intake of dairy products for breakfast was noticed in the youngest participants while statistically higher daily intake of vegetables was noticed in participants aged 4 to 5 years. The Portuguese Eat Mediterranean (EM) programme demonstrated that participants aged from 6 to 9 years old (68.9%) were the most adherent to the MeDi, whereas lower adherence to the MeDi was found in children aged 2 to 5 years (59.0%) and the lowest adherence was found for adolescents older than 15 years (46.0%) [23]. If we consider that most of our study participants were enrolled in a 10 h kindergarten program that provides breakfast and lunch as well as morning and afternoon snacks, it could be said that the diet of these children was uniform and, therefore, age-related differences should be minimal. Additionally, food fussiness (picky eating) and food neophobia are characteristic for children aged 2 to 5 years [24]. The most common food group that causes aversion is vegetables, because of their bitter taste and unique smell [25].

No statistically significant differences were found in the adherence to the total KIDMED score regarding the z-score values, except a borderline significance for fish consumption two or three times per week in underweight children and dairy consumption twice or more per day in overweight and obese children. In contrast to our results, previous studies found that a low adherence to the MeDi is associated with a high prevalence of overweight and obesity in children from the Mediterranean countries of the European Union [26,27,28]. Fish consumption was inversely associated with the z-score, which can be explained by the lower energy value of fish, as well as higher vegetable intake that is traditionally offered as a side dish to fish. Nevertheless, the evidence presented in the overview by Naveed et al. suggests that a diet rich in fish, vegetables and fruit, especially berries, is linked to better cognitive function and academic performance in children and adolescents [29]. Regarding dairy products intake and obesity rates, Dougkas et al. demonstrated that milk and dairy products do not have an impact on the development of childhood obesity, despite being a significant contribution to children’s nutrient intake. Although there may be some mechanisms that could explain the connection between dairy intake and adiposity based on appetite, lipid absorption and even intestinal microflora, there is still no evidence that suggests benefits of limiting dairy products for the purpose of preventing obesity in this particular population [30].

No significant correlations were determined between the total KIDMED score and WC as well as MUAC, unlike EnKid [14] and LLAL [31] studies, where the KIDMED score was inversely correlated to the WC. Regarding individual components of the KIDMED questionnaire, significant negative correlations were noticed for both WC and MUAC and vegetable consumption, as well as WC and the second daily fruit serving.

There are some limitations to this study. The KIDMED questionnaire was self-administered by the parents and data about household income and parental education are lacking. In addition, the study was conducted in one urban area but included a high number of age specific participants which is why the obtained results do not reflect the adherence to the MeDi for the entire Split-Dalmatia County. Further research should include participants from a larger geographical region, as well as a wider age range, to assess compliance to the MeDi principles.

## 5. Conclusions

The results of this study conducted with preschool children showed low adherence to the principles of the Mediterranean diet, which is characterized by a high presence of plant-based food as well as fish and dairy products. As such, the MeDi has been shown to have beneficial effects on body composition and obesity prevention in all age groups, which further emphasizes the importance of the timely implementation of this dietary pattern in the pediatric population, especially now, when the childhood obesity epidemic has reached its peak. Healthy eating habits play a major role in the growth and development of children, but also in preventing the development of chronic diseases in later life. Considering the favourable effects of the MeDi on overall health, further intervention studies could be useful for educating children and their caregivers about the benefits of healthy eating habits and of adopting them to reduce the risk of developing chronic non-communicable diseases in this population.

## Figures and Tables

**Table 1:** Basic characteristic and anthropometric measurements of the study population.

Sex [*n* (%)]	
Boys	310 (51.8)
Girls	288 (48.2)
Age (years) [Mean (SD)]	5 (1)
Age groups [*n* (%)]	
<4 years	15 (2.5)
4–5 years	290 (48.5)
6–7 years	293 (49)
Body height (m) [Mean (SD)]	1.17 (0.09)
Body weight (kg) [Median (IQR)] 12–40.5	21.2 (18.6–24.3)
Waist circumference (cm) [Median (IQR)] 43–76	54 (51–56)
Mid-upper arm circumference (cm) [Median (IQR)] 13–24	17.7 (16.5–19)
z-score [Median (IQR)]	0.25 (−0.40–0.92)
Nutritional status regarding z-score [*n* (%)]	
≤−1.04	54 (9)
>−1.04, <1.04	420 (70.2)
≥1.04	124 (20.7)
Supplements [*n* (%)]	124 (20.7)
Food allergies [*n* (%)]	>35 (5.9)
Number of meals [*n* (%)]	
3–4/day	117 (19.6)
4–5/day	396 (66.2)
>5/day	85 (14.2)
Length of kindergarten stay [*n* (%)]	
Half a day programme (5 h)	8 (1.3)
Half a day programme (6 h)	53 (8.9)
Half a day programme (6 h; lunch included)	19 (3.2)
Full day (10 h)	518 (86.6)

**Table 2:** Correlation between KIDMED index and waist and mid-upper arm circumference (only statistically significant values shown).

	WC	MUAC
Second serving of fruit daily	−0.085 (0.04 *)	−0.050 (0.23 *)
Fresh or cooked vegetables > 1/day	−0.091 (0.03 *)	−0.101 (0.01 *)

Abbreviations: WC—waist circumference, MUAC—mid-upper arm circumference * *p-*values were obtained with Point biserial correlation.

## Data Availability

Raw data can be found at corresponding authors via e-mail: elakolak93@gmail.com (E.K.); dorabucan@gmail.com (D.B.N.).

## Supplementary Materials

The following are available online at https://www.mdpi.com/article/10.3390/nu13124252/s1, Table S1: An example of a menu from kindergartens.
