# Supplementary material for: Anthropometric Parameters and Mediterranean Diet Adherence in Preschool Children in Split-Dalmatia County, Croatia—Are They Related?
Source: Nutrients. 2021 Nov 26;13(12):4252. doi: 10.3390/nu13124252 (PMC8706144; doi:10.3390/nu13124252)
Supplement: Supplementary file 1 [file nutrients-13-04252-s001.zip › nutrients-1485581-supplementary.pdf]

Table S1. An example of a menu from kindergartens.

**Full day kindergarten programme menu**				
	**Breakfast**	**Morning snack**	**Lunch**	**Afternoon snack**
**Monday**	milk, chocolate hazelnut spread on a white bread	apple or other seasonal fruit	beef and potato stew, lettuce, bread; muffin	chocolate pudding
**Tuesday**	cocoa, butter and rose-hip jam on a white bread	tangerine/orange	pumpkin soup, roast chicken breasts with cooked cabbage and potatoes (olive oil), white bread	probiotic yogurt
**Wednesday**	milk, gouda cheese with butter on white bread	pear or other seasonal fruit	lentil stew with meatballs, white bread	berry fruit tea with lemon and honey
**Thursday**	: fruit tea, cheese spread (30% milk fat) on a white bread	apple or other seasonal fruit	soup with millet, turkey in sauce with polenta, beetroot salad, bread; marble cake	milk, cornflakes
**Friday**	chocolate milk, butter with honey on a whole grain bread	banana	fried sardines with cooked chard and potatoes (olive oil), whole grain bread	semolina cooked with milk and chocolate

**- Five-hour and six-hour kindergarten programme –**		
	**First snack**	**Second snack**
**Monday** :	: milk, chocolate hazenut spread on a white bread	apple or other seasonal fruit
**Tuesday**	cocoa, butter and rose-hip jam on a white bread	tangerine/orange
**Wednesday**	milk, gouda cheese with butter on a white bread	pear or other seasonal fruit
**Thursday**	: fruit tea, cheese spread (30% milk fat) on a white bread	apple or other seasonal fruit
**Friday**	chocolate milk, butter and honey on a whole grain bread	banana
